# 4,5-Dicarb­oxy­naphthalene-1,8-dicarb­oxy­lic anhydride–1,10-phenanthroline (1/1)

**DOI:** 10.1107/S1600536811002492

**Published:** 2011-01-22

**Authors:** Xiang-Yang Wu, Xiang-Jun Xu, Xiang-Cheng Wang

**Affiliations:** a School of the Environment, Jiangsu University, Zhenjiang 212013, People’s Republic of China; b School of Chemistry and Chemical Engineering, Jiangsu University, Zhenjiang 212013, People’s Republic of China

## Abstract

In the crystal structure of the title 1:1 adduct, C_12_H_8_N_2_·C_14_H_6_O_7_, the carboxyl groups are involved in inter­molecular O—H⋯O hydrogen bonds, which link the mol­ecules into centrosymmetric dimers. These dimers are further linked by inter­molecular O—H⋯N hydrogen bonds. C—H⋯O inter­actions also occur between the 1,10-phenanthroline (phen) and 4,5-dicarb­oxy­naphthalene-1,8-dicarb­oxy­lic anhydride (H_2_NTC) mol­ecules. In addition, the crystal structure exhibits π–π inter­actions of the phen⋯phen and H_2_NTC⋯H_2_NTC types with centroid–centroid distances of 3.579 (3) and 3.774 (3) Å, respectively.

## Related literature

For background to the importance of 1,4,5,8-naphthalene­tetra­carb­oxy­lic acid and 1,10-phenanthroline, see: Chen *et al.* (2005 ▶); Che *et al.* (2006 ▶).
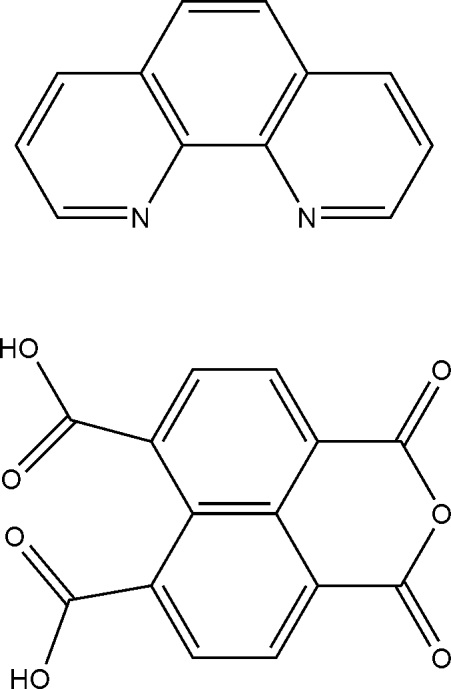


## Experimental

### 

#### Crystal data

C_12_H_8_N_2_·C_14_H_6_O_7_
*M* *_r_* = 466.39
Triclinic, 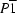

*a* = 9.0189 (5) Å
*b* = 10.1588 (7) Å
*c* = 11.2140 (8) Å
α = 104.267 (6)°
β = 92.278 (5)°
γ = 101.256 (5)°
*V* = 972.42 (11) Å^3^
*Z* = 2
Cu *K*α radiation
μ = 0.99 mm^−1^
*T* = 293 K
0.35 × 0.25 × 0.2 mm

#### Data collection

Bruker SMART CCD area-detector diffractometer
Absorption correction: multi-scan (*SADABS*; Bruker, 2002 ▶) *T*_min_ = 0.858, *T*_max_ = 1.000
6756 measured reflections
3416 independent reflections
2679 reflections with *I* > 2σ(*I*)
*R*_int_ = 0.061

#### Refinement

*R*[*F* ^2^ > 2σ(*F* ^2^)] = 0.056
*wR*(*F* ^2^) = 0.159
*S* = 0.99
3416 reflections
316 parameters
H-atom parameters constrained
Δρ_max_ = 0.49 e Å^−3^
Δρ_min_ = −0.41 e Å^−3^

### 

Data collection: *SMART* (Bruker, 2002 ▶); cell refinement: *SAINT* (Bruker, 2002 ▶); data reduction: *SAINT*; program(s) used to solve structure: *SHELXS97* (Sheldrick, 2008 ▶); program(s) used to refine structure: *SHELXL97* (Sheldrick, 2008 ▶); molecular graphics: *SHELXTL* (Sheldrick, 2008 ▶) and *DIAMOND* (Brandenburg, 1998 ▶); software used to prepare material for publication: *SHELXTL*.

## Supplementary Material

Crystal structure: contains datablocks global, I. DOI: 10.1107/S1600536811002492/lx2183sup1.cif

Structure factors: contains datablocks I. DOI: 10.1107/S1600536811002492/lx2183Isup2.hkl

Additional supplementary materials:  crystallographic information; 3D view; checkCIF report

## Figures and Tables

**Table 1:** Hydrogen-bond geometry (Å, °)

*D*—H⋯*A*	*D*—H	H⋯*A*	*D*⋯*A*	*D*—H⋯*A*
O2—H2*A*⋯N1^i^	0.82	1.97	2.683 (2)	144
O4—H4⋯O1^ii^	0.82	1.69	2.4637 (18)	158
C2—H2⋯O5^iii^	0.93	2.59	3.481 (3)	161
C8—H8⋯O3^ii^	0.93	2.57	3.312 (3)	137
C10—H10⋯O4^iv^	0.93	2.42	3.258 (3)	150

Symmetry codes: (i) 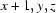; (ii) 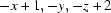; (iii) 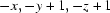; (iv) 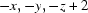.

**Fig. 1.**
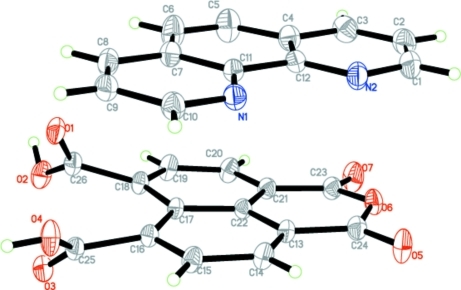
The molecular structure of the title compound with the atom numbering scheme. Displacement ellipsoids are drawn at the 30% probability level. H atoms are presented as a small spheres of arbitrary radius.

**Fig. 2.**
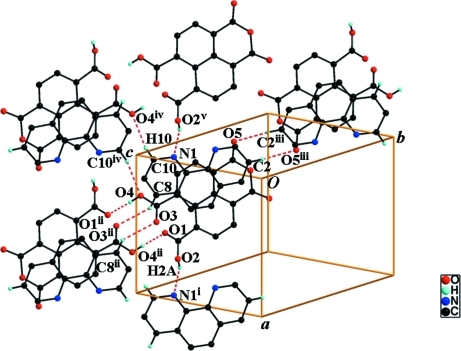
A view of the hydrogen bond and C–H···O interactions (dotted lines) in the crystal structure of the title compound. [Symmetry codes: (i) x + 1, y, z; (ii) - x + 1, - y, - z + 2; (iii) - x, - y + 1, - z + 1; (iv) - x, - y, - z + 2; (v) x - 1, y, z.]

**Fig. 3.**
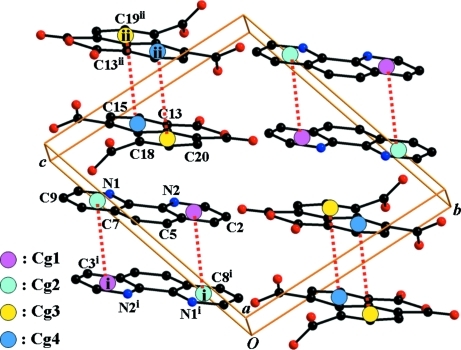
A view of π-π interactions (dotted lines) in the unit cell of the title compound. [Symmetry codes: (i) - x, - y, - z + 1; (ii) -x + 1, - y + 1, - z + 2.]

## supplementary crystallographic information

### Comment

1,4,5,8-Naphthalenetetracarboxylic acid (H_4_NTC) is of special interest since its high symmetry and large π-conjugated structure can allow to construct molecular assemblies with novel structure motifs and physical properties (Chen *et al.*, 2005). The 1,10-phenanthroline (phen) has been widely used to build novel supramolecular architectures through aromatic π..π interactions (Che *et al.*, 2006). We report herein on the crystal structure of the title compound (Fig. 1).

In the crystal packing (Fig. 2), the carboxyl groups are involved in intermolecular O–H···O hydrogen bonds, which link the molecules into centrosymmetric dimers. These dimers are further linked by an intermolecular O–H···N hydrogen bond. There are also C–H···O interactions between the phen and H_2_NTC (Table 1). In addition, the crystal structure exhibit the π-π interactions between the phen···phen and H_2_NTC···H_2_NTC, respectively. The π-π interaction distance (Cg1-to-Cg2^i^) between the phen···phen is 3.579 (3) Å, and the π-π interaction distance (Cg3-to-Cg4^ii^) between the H_2_NTC···H_2_NTC is 3.774 (3) Å (Fig. 3). Cg1, Cg2, Cg3 and Cg4 are centroids of the N2-C2, N1-C7, C18-C20 and C13-C15 ring, respectively.

### Experimental

The reagents, purchased from standard commercial sources and without further purification, were 1,4,5,8-naphthalenetetracarboxylic acid and 1,10-phenanthroline. A mixture of H_4_NTC (0.0304 g, 0.10 mmol), phen (0.018 g, 0.10 mmol) and water (10 mL) in a 25 mL Teflon-lined stainless steel autoclave was heated for 3 d at 433 K under autogenous pressure and cooled to room temperature. Yellow block crystals were obtained.

### Refinement

All H atoms on C atoms were positioned geometrically and refined as riding atoms, with C–H = 0.93 Å and *U*_iso_= 1.2 *U*_eq_(C). The hydroxyl H atoms were located in a difference Fourier map, and were refined with suitable O–H distance restraint; *U*_iso_ = 1.5 *U*_eq_(O).

### Crystal data

**Table:**

C_12_H_8_N_2_·C_14_H_6_O_7_	*Z* = 2
*M**_r_* = 466.39	*F*(000) = 480
Triclinic, *P*1	*D*_x_ = 1.593 Mg m^−^^3^
Hall symbol: -P 1	Cu *K*α radiation, λ = 1.54184 Å
*a* = 9.0189 (5) Å	Cell parameters from 3294 reflections
*b* = 10.1588 (7) Å	θ = 4.1–67.0°
*c* = 11.2140 (8) Å	µ = 0.99 mm^−^^1^
α = 104.267 (6)°	*T* = 293 K
β = 92.278 (5)°	Block, yellow
γ = 101.256 (5)°	0.35 × 0.25 × 0.2 mm
*V* = 972.42 (11) Å^3^	

### Data collection

**Table:**

Bruker SMART CCD area-detector diffractometer	3416 independent reflections
Radiation source: fine-focus sealed tube	2679 reflections with *I* > 2σ(*I*)
graphite	*R*_int_ = 0.061
φ and ω scans	θ_max_ = 67.1°, θ_min_ = 4.1°
Absorption correction: multi-scan (*SADABS*; Bruker, 2002)	*h* = −7→10
*T*_min_ = 0.858, *T*_max_ = 1.000	*k* = −12→12
6756 measured reflections	*l* = −13→13

### Refinement

**Table:**

Refinement on *F*^2^	Primary atom site location: structure-invariant direct methods
Least-squares matrix: full	Secondary atom site location: difference Fourier map
*R*[*F*^2^ > 2σ(*F*^2^)] = 0.056	Hydrogen site location: difference Fourier map
*wR*(*F*^2^) = 0.159	H-atom parameters constrained
*S* = 0.99	*w* = 1/[σ^2^(*F*_o_^2^) + (0.1188*P*)^2^] where *P* = (*F*_o_^2^ + 2*F*_c_^2^)/3
3416 reflections	(Δ/σ)_max_ < 0.001
316 parameters	Δρ_max_ = 0.49 e Å^−^^3^
0 restraints	Δρ_min_ = −0.41 e Å^−^^3^

### Special details

**Table:**

Geometry. All esds (except the esd in the dihedral angle between two l.s. planes) are estimated using the full covariance matrix. The cell esds are taken into account individually in the estimation of esds in distances, angles and torsion angles; correlations between esds in cell parameters are only used when they are defined by crystal symmetry. An approximate (isotropic) treatment of cell esds is used for estimating esds involving l.s. planes.
Refinement. Refinement of F^2^ against ALL reflections. The weighted R-factor wR and goodness of fit S are based on F^2^, conventional R-factors R are based on F, with F set to zero for negative F^2^. The threshold expression of F^2^ > 2sigma(F^2^) is used only for calculating R-factors(gt) etc. and is not relevant to the choice of reflections for refinement. R-factors based on F^2^ are statistically about twice as large as those based on F, and R- factors based on ALL data will be even larger.

### Fractional atomic coordinates and isotropic or equivalent isotropic displacement parameters (Å^2^)

**Table:**

	*x*	*y*	*z*	*U*_iso_*/*U*_eq_	
O1	0.55753 (16)	0.06531 (14)	0.84023 (14)	0.0425 (4)	
O2	0.79210 (15)	0.19823 (17)	0.87789 (16)	0.0469 (4)	
H2A	0.8273	0.1546	0.8181	0.070*	
O3	0.57213 (15)	0.23703 (14)	1.08928 (14)	0.0399 (4)	
O4	0.33967 (16)	0.10420 (17)	1.07563 (16)	0.0471 (4)	
H4	0.3822	0.0657	1.1189	0.071*	
O5	0.04091 (17)	0.56071 (18)	0.81155 (17)	0.0538 (5)	
O6	0.23485 (16)	0.57884 (15)	0.70001 (13)	0.0415 (4)	
O7	0.4382 (2)	0.6322 (2)	0.60562 (17)	0.0583 (5)	
N1	−0.00536 (18)	0.06070 (18)	0.76513 (16)	0.0364 (4)	
N2	−0.0387 (2)	0.2209 (2)	0.60381 (17)	0.0437 (4)	
C1	−0.0510 (3)	0.2947 (3)	0.5239 (2)	0.0510 (6)	
H1	−0.1358	0.3342	0.5238	0.061*	
C2	0.0537 (3)	0.3179 (2)	0.4397 (2)	0.0512 (6)	
H2	0.0392	0.3718	0.3859	0.061*	
C3	0.1778 (3)	0.2602 (3)	0.4375 (2)	0.0498 (6)	
H3	0.2494	0.2740	0.3819	0.060*	
C4	0.1968 (2)	0.1796 (2)	0.5201 (2)	0.0421 (5)	
C5	0.3233 (3)	0.1145 (3)	0.5236 (3)	0.0587 (7)	
H5	0.3993	0.1286	0.4717	0.070*	
C6	0.3346 (3)	0.0337 (3)	0.6003 (3)	0.0567 (7)	
H6	0.4176	−0.0082	0.5995	0.068*	
C7	0.2219 (2)	0.0106 (2)	0.6831 (2)	0.0385 (5)	
C8	0.2284 (2)	−0.0737 (2)	0.7640 (2)	0.0422 (5)	
H8	0.3086	−0.1189	0.7647	0.051*	
C9	0.1178 (2)	−0.0901 (2)	0.8420 (2)	0.0405 (5)	
H9	0.1211	−0.1472	0.8949	0.049*	
C10	0.0006 (2)	−0.0202 (2)	0.84104 (19)	0.0395 (5)	
H10	−0.0749	−0.0301	0.8942	0.047*	
C11	0.1000 (2)	0.0779 (2)	0.68408 (18)	0.0325 (4)	
C12	0.0848 (2)	0.1632 (2)	0.60101 (18)	0.0348 (4)	
C13	0.2358 (2)	0.44630 (19)	0.85406 (18)	0.0314 (4)	
C14	0.1623 (2)	0.3913 (2)	0.9408 (2)	0.0375 (5)	
H14	0.0672	0.4080	0.9601	0.045*	
C15	0.2311 (2)	0.3098 (2)	1.00005 (19)	0.0361 (4)	
H15	0.1822	0.2756	1.0611	0.043*	
C16	0.3691 (2)	0.27875 (19)	0.97076 (17)	0.0303 (4)	
C25	0.4381 (2)	0.20230 (19)	1.05024 (17)	0.0316 (4)	
C26	0.6530 (2)	0.1802 (2)	0.85624 (19)	0.0350 (4)	
C17	0.4462 (2)	0.32898 (18)	0.87661 (17)	0.0282 (4)	
C18	0.5859 (2)	0.2975 (2)	0.83327 (18)	0.0330 (4)	
C19	0.6576 (2)	0.3638 (2)	0.7515 (2)	0.0417 (5)	
H19	0.7522	0.3482	0.7293	0.050*	
C20	0.5921 (2)	0.4540 (2)	0.7011 (2)	0.0419 (5)	
H20	0.6428	0.4974	0.6459	0.050*	
C21	0.4528 (2)	0.4785 (2)	0.73298 (18)	0.0338 (4)	
C22	0.3780 (2)	0.41784 (18)	0.82119 (17)	0.0289 (4)	
C23	0.3816 (2)	0.5682 (2)	0.67520 (19)	0.0389 (5)	
C24	0.1622 (2)	0.5316 (2)	0.79219 (19)	0.0371 (5)	

### Atomic displacement parameters (Å^2^)

**Table:**

	*U*^11^	*U*^22^	*U*^33^	*U*^12^	*U*^13^	*U*^23^
O1	0.0442 (8)	0.0336 (8)	0.0555 (9)	0.0109 (6)	−0.0002 (7)	0.0212 (7)
O2	0.0352 (8)	0.0528 (9)	0.0640 (10)	0.0208 (7)	0.0146 (7)	0.0256 (8)
O3	0.0383 (8)	0.0401 (8)	0.0454 (8)	0.0087 (6)	−0.0018 (6)	0.0190 (6)
O4	0.0385 (8)	0.0480 (9)	0.0698 (10)	0.0107 (7)	0.0066 (7)	0.0417 (8)
O5	0.0447 (9)	0.0638 (11)	0.0712 (11)	0.0291 (8)	0.0095 (8)	0.0372 (9)
O6	0.0459 (8)	0.0451 (8)	0.0428 (8)	0.0162 (6)	0.0012 (6)	0.0240 (7)
O7	0.0721 (11)	0.0672 (11)	0.0600 (11)	0.0310 (9)	0.0226 (9)	0.0462 (9)
N1	0.0358 (9)	0.0413 (9)	0.0357 (9)	0.0130 (7)	0.0040 (7)	0.0128 (7)
N2	0.0476 (10)	0.0500 (11)	0.0421 (10)	0.0231 (8)	0.0037 (8)	0.0177 (8)
C1	0.0599 (14)	0.0536 (14)	0.0475 (13)	0.0245 (11)	−0.0022 (11)	0.0190 (11)
C2	0.0679 (15)	0.0445 (12)	0.0453 (12)	0.0111 (11)	−0.0047 (11)	0.0214 (10)
C3	0.0553 (14)	0.0497 (13)	0.0477 (13)	0.0050 (11)	0.0045 (10)	0.0240 (11)
C4	0.0403 (11)	0.0440 (12)	0.0455 (12)	0.0088 (9)	0.0037 (9)	0.0180 (10)
C5	0.0470 (13)	0.0779 (18)	0.0676 (16)	0.0252 (12)	0.0226 (12)	0.0373 (14)
C6	0.0441 (12)	0.0761 (17)	0.0690 (16)	0.0325 (12)	0.0222 (11)	0.0359 (14)
C7	0.0340 (10)	0.0409 (11)	0.0440 (11)	0.0122 (8)	0.0036 (8)	0.0140 (9)
C8	0.0400 (11)	0.0407 (11)	0.0508 (12)	0.0163 (9)	0.0001 (9)	0.0158 (10)
C9	0.0468 (11)	0.0366 (11)	0.0409 (11)	0.0098 (9)	0.0003 (9)	0.0150 (9)
C10	0.0431 (11)	0.0430 (11)	0.0377 (10)	0.0129 (9)	0.0061 (8)	0.0172 (9)
C11	0.0312 (9)	0.0335 (10)	0.0340 (10)	0.0093 (8)	0.0009 (7)	0.0096 (8)
C12	0.0369 (10)	0.0350 (10)	0.0338 (10)	0.0095 (8)	−0.0013 (8)	0.0104 (8)
C13	0.0324 (9)	0.0296 (9)	0.0365 (10)	0.0104 (7)	0.0018 (8)	0.0133 (8)
C14	0.0357 (10)	0.0390 (11)	0.0463 (11)	0.0155 (9)	0.0108 (9)	0.0199 (9)
C15	0.0380 (10)	0.0357 (10)	0.0425 (11)	0.0129 (8)	0.0117 (8)	0.0199 (9)
C16	0.0321 (9)	0.0280 (9)	0.0344 (10)	0.0089 (7)	0.0033 (8)	0.0129 (8)
C25	0.0346 (10)	0.0303 (9)	0.0345 (10)	0.0104 (8)	0.0055 (8)	0.0136 (8)
C26	0.0353 (10)	0.0377 (11)	0.0399 (10)	0.0141 (8)	0.0104 (8)	0.0187 (9)
C17	0.0281 (9)	0.0258 (8)	0.0342 (9)	0.0072 (7)	0.0029 (7)	0.0133 (7)
C18	0.0333 (10)	0.0310 (10)	0.0405 (10)	0.0095 (8)	0.0067 (8)	0.0173 (8)
C19	0.0371 (10)	0.0446 (12)	0.0554 (13)	0.0162 (9)	0.0179 (9)	0.0272 (10)
C20	0.0463 (12)	0.0412 (11)	0.0493 (12)	0.0142 (9)	0.0178 (9)	0.0264 (10)
C21	0.0403 (10)	0.0323 (10)	0.0337 (10)	0.0102 (8)	0.0063 (8)	0.0152 (8)
C22	0.0322 (9)	0.0264 (9)	0.0303 (9)	0.0071 (7)	0.0016 (7)	0.0110 (7)
C23	0.0474 (12)	0.0385 (11)	0.0378 (11)	0.0144 (9)	0.0071 (9)	0.0185 (9)
C24	0.0388 (10)	0.0352 (10)	0.0415 (11)	0.0111 (8)	0.0007 (8)	0.0152 (9)

### Geometric parameters (Å, °)

**Table:**

O1—C26	1.276 (2)	C7—C11	1.403 (3)
O2—C26	1.237 (2)	C8—C9	1.365 (3)
O2—H2A	0.8200	C8—H8	0.9300
O3—C25	1.222 (2)	C9—C10	1.384 (3)
O4—C25	1.295 (2)	C9—H9	0.9300
O4—H4	0.8200	C10—H10	0.9300
O5—C24	1.202 (3)	C11—C12	1.439 (3)
O6—C24	1.380 (3)	C13—C14	1.369 (3)
O6—C23	1.382 (3)	C13—C22	1.412 (3)
O7—C23	1.201 (3)	C13—C24	1.469 (3)
N1—C10	1.327 (3)	C14—C15	1.396 (3)
N1—C11	1.357 (3)	C14—H14	0.9300
N2—C1	1.317 (3)	C15—C16	1.376 (3)
N2—C12	1.354 (3)	C15—H15	0.9300
C1—C2	1.391 (4)	C16—C17	1.430 (3)
C1—H1	0.9300	C16—C25	1.507 (2)
C2—C3	1.359 (4)	C26—C18	1.508 (3)
C2—H2	0.9300	C17—C22	1.429 (2)
C3—C4	1.405 (3)	C17—C18	1.434 (3)
C3—H3	0.9300	C18—C19	1.376 (3)
C4—C12	1.398 (3)	C19—C20	1.396 (3)
C4—C5	1.429 (3)	C19—H19	0.9300
C5—C6	1.341 (3)	C20—C21	1.372 (3)
C5—H5	0.9300	C20—H20	0.9300
C6—C7	1.428 (3)	C21—C22	1.416 (3)
C6—H6	0.9300	C21—C23	1.466 (3)
C7—C8	1.399 (3)		
			
C26—O2—H2A	109.5	C14—C13—C24	119.01 (17)
C25—O4—H4	109.5	C22—C13—C24	120.29 (17)
C24—O6—C23	123.76 (15)	C13—C14—C15	119.51 (18)
C10—N1—C11	122.47 (17)	C13—C14—H14	120.2
C1—N2—C12	116.5 (2)	C15—C14—H14	120.2
N2—C1—C2	124.6 (2)	C16—C15—C14	121.89 (18)
N2—C1—H1	117.7	C16—C15—H15	119.1
C2—C1—H1	117.7	C14—C15—H15	119.1
C3—C2—C1	118.6 (2)	C15—C16—C17	120.23 (16)
C3—C2—H2	120.7	C15—C16—C25	116.49 (16)
C1—C2—H2	120.7	C17—C16—C25	123.04 (16)
C2—C3—C4	119.4 (2)	O3—C25—O4	125.51 (17)
C2—C3—H3	120.3	O3—C25—C16	121.47 (16)
C4—C3—H3	120.3	O4—C25—C16	112.93 (16)
C12—C4—C3	117.3 (2)	O2—C26—O1	125.89 (18)
C12—C4—C5	119.7 (2)	O2—C26—C18	119.39 (17)
C3—C4—C5	123.1 (2)	O1—C26—C18	114.43 (16)
C6—C5—C4	121.4 (2)	C22—C17—C16	117.17 (16)
C6—C5—H5	119.3	C22—C17—C18	117.40 (17)
C4—C5—H5	119.3	C16—C17—C18	125.43 (16)
C5—C6—C7	121.4 (2)	C19—C18—C17	120.01 (17)
C5—C6—H6	119.3	C19—C18—C26	114.89 (17)
C7—C6—H6	119.3	C17—C18—C26	124.54 (16)
C8—C7—C11	118.72 (19)	C18—C19—C20	121.70 (18)
C8—C7—C6	123.41 (19)	C18—C19—H19	119.2
C11—C7—C6	117.86 (19)	C20—C19—H19	119.2
C9—C8—C7	120.47 (19)	C21—C20—C19	119.95 (19)
C9—C8—H8	119.8	C21—C20—H20	120.0
C7—C8—H8	119.8	C19—C20—H20	120.0
C8—C9—C10	118.9 (2)	C20—C21—C22	120.37 (18)
C8—C9—H9	120.6	C20—C21—C23	119.60 (18)
C10—C9—H9	120.6	C22—C21—C23	120.03 (17)
N1—C10—C9	120.8 (2)	C13—C22—C21	119.40 (16)
N1—C10—H10	119.6	C13—C22—C17	120.38 (17)
C9—C10—H10	119.6	C21—C22—C17	120.23 (17)
N1—C11—C7	118.59 (18)	O7—C23—O6	116.28 (18)
N1—C11—C12	120.00 (17)	O7—C23—C21	125.9 (2)
C7—C11—C12	121.40 (18)	O6—C23—C21	117.81 (17)
N2—C12—C4	123.60 (19)	O5—C24—O6	116.34 (17)
N2—C12—C11	118.22 (18)	O5—C24—C13	125.90 (19)
C4—C12—C11	118.17 (18)	O6—C24—C13	117.71 (17)
C14—C13—C22	120.67 (17)		

### Hydrogen-bond geometry (Å, °)

**Table:**

*D*—H···*A*	*D*—H	H···*A*	*D*···*A*	*D*—H···*A*
O2—H2A···N1^i^	0.82	1.97	2.683 (2)	144
O4—H4···O1^ii^	0.82	1.69	2.4637 (18)	158
C2—H2···O5^iii^	0.93	2.59	3.481 (3)	161
C8—H8···O3^ii^	0.93	2.57	3.312 (3)	137
C10—H10···O4^iv^	0.93	2.42	3.258 (3)	150

Symmetry codes: (i) *x*+1, *y*, *z*; (ii) −*x*+1, −*y*, −*z*+2; (iii) −*x*, −*y*+1, −*z*+1; (iv) −*x*, −*y*, −*z*+2.

## References

[bb1] Brandenburg, K. (1998). *DIAMOND* Crystal Impact GbR, Bonn, Germany.

[bb2] Bruker (2002). *SADABS*, *SAINT* and *SMART* Bruker AXS Inc., Madison, Wisconsin, USA.

[bb3] Che, G.-B., Xu, Z.-L. & Liu, C.-B. (2006). *Acta Cryst.* E**62**, m1370–m1372.

[bb4] Chen, L.-F., Zhang, C., Song, L.-J. & Ju, Z.-F. (2005). *Inorg. Chem. Commun.* **8**, 555–558.

[bb5] Sheldrick, G. M. (2008). *Acta Cryst.* A**64**, 112–122. 10.1107/S0108767307043930 18156677

